# Escholtzia Californica

**Published:** 1891-02-28

**Authors:** 


### THERAPEUTICS.

### ESCHOLTZIA CALIFORNICA.

This plant belongs to the class *Papaveraceæ*. It has been investigated by Bardet and Adrian, of Paris, who find that it yields on examination a glucoside, an organic oil, and a small quantitiy of morphine (thirty to forty centigrammes in a kilogramme of the dried leaves, or about five or six grains to the pound). It has been employed as a sedative, especially for children. It was first suggested as a substitute for opium in children upon the erroneous supposition that it did not contain morphine. We have used it in a considerable number of cases as a hypnotic, and its results were favourable, but as its action appears to be due simply to the traces of morphine it contains it will propably prove more interesting than useful, and certainly not to be advocated as a hypnotic for children.

Ter-Zakariant's experiments seem to show that the alcoholic extract of the plant, when administered in small doses, appears to arrest the cerebral functions.

